# Network-Based Gene Expression Biomarkers for Cold and Heat Patterns of Rheumatoid Arthritis in Traditional Chinese Medicine

**DOI:** 10.1155/2012/203043

**Published:** 2012-03-22

**Authors:** Cheng Lu, Xuyan Niu, Cheng Xiao, Gao Chen, Qinglin Zha, Hongtao Guo, Miao Jiang, Aiping Lu

**Affiliations:** ^1^Institute of Basic Research in Clinical Medicine, China Academy of Chinese Medical Science, No. 16, Nanxiaojie, Dongzhimennei, Beijing 100700, China; ^2^Institute of Clinical Medicine, China-Japan Hospital, Beijing 100029, China; ^3^School of Life Science, Hubei University, Wuhan, Hubei 430062, China; ^4^National Research Center of TCM, Jiangxi University of Chinese Medicine, Nanchang, Jiangxi 330006, China; ^5^E-Institute of Shanhai Municipal Education Commission, Shanghai TCM University, Shanghai 201203, China

## Abstract

In Traditional Chinese Medicine (TCM), patients with Rheumatoid Arthritis (RA) can be classified into two main patterns: cold-pattern and heat-pattern. This paper identified the network-based gene expression biomarkers for both cold- and heat-patterns of RA. Gene expression profilings of CD4+ T cells from cold-pattern RA patients, heat-pattern RA patients, and healthy volunteers were obtained using microarray. The differentially expressed genes and related networks were explored using DAVID, GeneSpring software, and the protein-protein interactions (PPI) method. EIF4A2, CCNT1, and IL7R, which were related to the up-regulation of cell proliferation and the Jak-STAT cascade, were significant gene biomarkers of the TCM cold pattern of RA. PRKAA1, HSPA8, and LSM6, which were related to fatty acid metabolism and the I-**κ**B kinase/NF-**κ**B cascade, were significant biomarkers of the TCM heat-pattern of RA. The network-based gene expression biomarkers for the TCM cold- and heat-patterns may be helpful for the further stratification of RA patients when deciding on interventions or clinical trials.

## 1. Introduction

Rheumatoid arthritis (RA) is a systemic autoimmune disease characterized by chronic inflammation throughout the body, particularly in diarthrodial joints [1, 2], and inflammatory cell infiltration, including activated CD4+ T cells in peripheral blood [3]. There is substantial evidence that CD4+ T cells play a key role in inflammatory processes in the pathogenesis of RA [4, 5] through their ability to stimulate the secretion of proinflammatory cytokines, which induces immunoglobulin production and matrix metalloproteinase secretion and causes osteoclastogenesis [6]. A hallmark pathological feature of RA is the infiltration and accumulation of T cells in the synovium of joints [7]. Because the shared epitope in human leukocyte antigen-DR genes is found in approximately 80% of RA patients, dysregulated CD4+ T-cell activation and function have been investigated based on available genetic predisposition evidence [8, 9]. 

Pattern classification, a key aspect of traditional Chinese medicine (TCM) therapeutic theory, is based on symptoms, tongue appearance, and pulse feelings [10]. The efficacy of TCM is based entirely on pattern differentiation because the pattern guides the prescription [11–13]. An increasing number of medical researchers are recognizing that the combination of disease diagnosis in biomedicine and pattern classification in TCM is essential for clinical practice. This model is commonly practiced in China because it produces positive clinical effects [14]. In many clinical studies that employed TCM pattern classification, desirable outcomes were achieved [15–23]. Moreover, the correlations between TCM patterns and biomedical parameters are receiving an increasing amount of attention during the mechanism exploration step of TCM pattern classification. Such correlations include the linkage between sex hormones and Kidney deficiency syndrome in chronic nephritis [24], the linkage between the C-reactive protein (CRP) and cold and hot syndromes in rheumatoid arthritis (RA) [25], the association between the Qi-Yin deficiency syndrome in type 2 diabetes mellitus (T2DM) patients with macroangiopathy and the apolipo-protein E (APOE) E4 and E3 genotypes [26], the correlation between the serum level of eosinophil cationic protein (ECP) in asthmatic patients and the development of “heat” syndrome manifestations [27], and the close relationship between decreases in skin electrical conductance and the severity of qi vacuity [28]. Therefore, the classification of different TCM patterns for specific diseases is important in both clinical practice and related basic research. 

RA patients can be classified into two main TCM patterns: the cold pattern and the heat pattern. Manifestations of the cold pattern are described as severe fixed pain in a joint or muscle, pain relief upon warming, severe pain when cooling, and a white tongue coating. In contrast, manifestations of the heat pattern are characterized by severe pain, hot, red, swollen and inflamed joints, pain relief upon cooling, severe pain upon warming, fever, thirst, restlessness, deep-colored urine, and a red tongue with a yellow coating [13, 29]. Our previous study showed that the effective rate of biomedical combination therapy was higher in patients with a cold pattern than in patients with a heat pattern (*P *< 0.01) [29]. These different responses to treatment led to the hypothesis that these patterns in TCM have their own specific markers. 

An important part of systems biology, microarrays provide a powerful method for cataloging genes that are affected or mediated by a given disease [30, 31], such as RA [32, 33]. Increasingly informative software programs that aid in the translation of these catalogues of genes from microarray experiments into an understanding of disease pathology are available. Biological systems are both redundant and highly networked. As a consequence, many functionally interrelated genes tend to be affected when the pathological effects on a given pathway are significant. These pathways can be characterized through a secondary analysis of differentially regulated gene sets using clustering and networking algorithms and a visual analysis of gene function. Ma T coupled the classical TCM cold syndrome with methods from systems biology to explore the macro-and microsystems biology frameworks of TCM syndromes. The results of this study indicate that genes related to the cold syndrome play an essential role in energy metabolism [34]. In a previous report, we used microarray technology to reveal gene expression profiles in CD4+ T cells and showed that 29 genes were differentially regulated in RA patients with cold and heat patterns [35]. We identified the differentially expressed genes in the cold and heat pattern RA patients. However, the comparisons between the cold-pattern RA patients and healthy persons and those between the heat-pattern RA patients and healthy persons may be more important in identifying the biomarkers of the cold and heat patterns. In the present study, we applied gene expression profiling of peripheral CD4+ T cells from cold-pattern RA patients, heat-pattern RA patients, and healthy volunteers to identify the differentially expressed genes and related networks for cold- and heat-pattern RA patients based on the differences from healthy persons and to further reveal the network-based biomarkers for the cold and heat patterns using the Database for Annotation, Visualization and Integrated Discovery (DAVID), the GeneSpring Software, and a PPI (protein-protein interactions) analysis. 

## 2. Materials and Methods

### 2.1. Patients

A total of 33 female RA patients from the China-Japan Friendship Hospital and 12 healthy female volunteers from the China Academy of Chinese Medical Science in Beijing, aged 18 to 70 years old, participated in the study. RA patients were eligible to participate if they had met the American College of Rheumatology (ACR) criteria for RA for at least one year, with a functional class of I, II, or III [36]. Patients were diagnosed and classified into either the heat pattern group or the cold pattern group according to TCM theory using a questionnaire, a tongue examination, and pulse diagnosis by 3 appointed TCM practitioners [13, 29]. Patients were included in the study only if the 3 practitioners reported consistent results. This ensured that all of the selected patients had typical manifestations of the heat or cold patterns according to TCM theory. Healthy females without any diagnosed diseases were included as controls.

Patients who had continuously received nonsteroidal anti-inflammatory corticosteroid drugs for more than 6 months or who had received these medicines within one month were excluded from the study. Patients with severe cardiovascular, lung, liver, kidney, mental, or blood system diseases and women who were pregnant, breast-feeding, or planning pregnancy in the next 8 months were excluded from the study. A complete joint function and biochemical function evaluation was available for all participants in the study. 

### 2.2. Sample Preparation

For the microarray, 8 mL of venous blood was collected in anticoagulation tubes from each of the 45 participants (12 patients with the heat pattern, 21 patients with the cold pattern, and 12 healthy volunteers) before breakfast. CD4+ T cells were extracted and purified using the RosetteSep Human CD4+ T Cell Enrichment Cocktail (StemCell Technologies, Inc., Vancouver, Canada). Total RNA was isolated from the CD4+ T cells using the Trizol extraction method (Invitrogen, Carlsbad, Canada), as described by the manufacturer. mRNA was amplified linearly using the MessageAmp*™* aRNA Kit (Ambion, Inc., Austin, USA), in accordance with the instructions of the manufacturer. cRNA was purified using an RNeasy Mini Kit (QIAGEN, Hilden, Germany) based on a standard procedure. 

### 2.3. Microarray Assay

A two-color whole Human Genome Microarray Kit, 4 × 44 K (Agilent Technologies) was used in this study. Microarray hybridizations were performed on labeled cRNAs. All arrays had the same labels: Cy3 for samples and Cy5 for controls. The arrays were incubated at 65°C for 17 h in Agilent's microarray hybridization chambers and subsequently washed according to the Agilent protocol. The arrays were scanned at a 5 *μ*m resolution using a Genepix 4000B scanner (Molecular Devices Corporation, Sunnyvale, CA). Auto-photomultiplier tube (PMT) gains were adjusted to obtain a ratio of Cy3 and Cy5 channel intensities. Scanned picture information was transformed into data using the GenePix Pro Microarray Image Analysis Software. One assay was completed for each sample, and biological replication was adopted to reduce the systematic sources of variation common in macroarray studies [37]. 

### 2.4. Statistics and Functional Analysis

#### 2.4.1. Microarray Statistics

All of the data were analyzed using the SAS9.1.3 statistical package (order no. 195557). The data were normalized to correct for technical variations among individual microarray hybridizations using the two-step procedure described in detail by Jarvis and colleagues. The signal intensity of each expressed gene was globally normalized (LOWESS) using the R statistics program [38]. Any ratio between two groups of more or less than 1 : 1.4 was taken as the differential gene expression criteria. Statistical significance was tested using the Student's *t*-test  (*P* < 0.05). Changes greater than 1.4-fold (cold pattern to control group or heat pattern to control group) were recorded as upregulations, and those less than 1.4-fold (cold pattern to control group or heat pattern to control group) were recorded as downregulations. Other fold changes for gene expression were recorded as normal expression. Changes in gene expression (1.4-fold change) were required in more than 50% of the patients. A chi-squared test was used for these comparisons (*P* < 0.05) and to identify similar and different genes in the cold and heat pattern groups of differentially expressed genes.

Gene assemblages were obtained using a principal components analysis and an iterated principal factor analysis. A cut-off value of 1.5 for the foreground and background signal ratios was used. The fluorescence ratio for each spot was log-transformed for normalization. A cluster analysis was performed using Cluster 3.0 and Tree View software. 

#### 2.4.2. GeneSpring Analysis

A global comparison of all cell lines was performed using GeneSpring GX v 7.3.1 (Agilent Technologies) and the gene annotations available in March 2009 to find differentially expressed genes in the majority of resistant cell lines. Triplicate samples for the two conditions (sensitive and resistant) in each of the seven cell lines (45 samples in total) were imported into one single experiment. The expression of each gene was calculated as the ratio of the value obtained for each condition relative to the control condition after data normalization. The data were filtered using the control strength, and a control value was calculated using the Cross-Gene Error Model on replicates based on the average base/proportional value. Measurements with higher control strengths are relatively more precise than measurements with lower control strengths. Genes that did not reach this value were discarded. Additional filtering was performed to identify the differentially expressed genes. We selected the genes that displayed a *P* value corrected by a false discovery rate (Benjamin and Hochberg FDR) of less than 0.05. 

Of the significantly differentially expressed RNA, only genes with a greater than 1.4-fold increase or 1.4-fold decrease in expression compared to the controls were used for further analysis. All microarray data in this study are in accordance with MIAME guidelines and have been deposited in the NCBI GEO database.

#### 2.4.3. Gene Ontology and KEGG Pathway Analysis Using DAVID

We used DAVID Bioinformatics Resources 6.7 (the Database for Annotation, Visualization, and Integrated Discovery) [39], “http://david.abcc.ncifcrf.gov/”, a comprehensive set of functional annotation tools for understanding the biological meaning behind large lists of genes, to obtain gene ontology and KEGG pathway information for differential genes between the cold pattern and control samples and between the heat pattern and control samples. Differentially expressed genes that were similar and different between the cold pattern and the control and between the heat pattern and the control were compared. In the analysis, the control was set at the 0.01 level for the number of false positives using two statistics: False Detection Rate (FDR) and Bonferroni correction (FWER). 

#### 2.4.4. Protein-Protein Interaction Analysis

PPIS are the basic skeleton for the self-organization and homeostasis of living organisms [40]. In this study, information on human PPI networks from significant genes was obtained from databases, including the BIND, BIOGRID, DIP, HPRD, IntAct, and MINT, and complemented with relationships that were parsed from the literature using Agilent Literature Search. The PPI network was visualized using the Cytoscape software [41].

We integrated the databases and networks and used an IPCA to analyze the characteristics of the networks. The IPCA algorithm can detect highly connected regions or clusters in the interactome network [42]. Interactomes with a score greater than 2.0 and at least four nodes were taken as significant predictions.

Gene ontology categories were further analyzed to identify the function of each highly connected region generated by the IPCA. The latest version of the Biological Network Gene Ontology (BINGO) tool was used to statistically evaluate groups of proteins with respect to the existing annotations of the Gene Ontology Consortium. The degree of functional enrichment for a given cluster was quantitatively assessed (*P* value) using a hypergeometric distribution implemented in the BINGO tool.

## 3. Results

### 3.1. Differential Gene Expression : RA with the Cold Pattern versus the Healthy Control and RA with the Heat Pattern versus the Healthy Control

**Figure 1:**
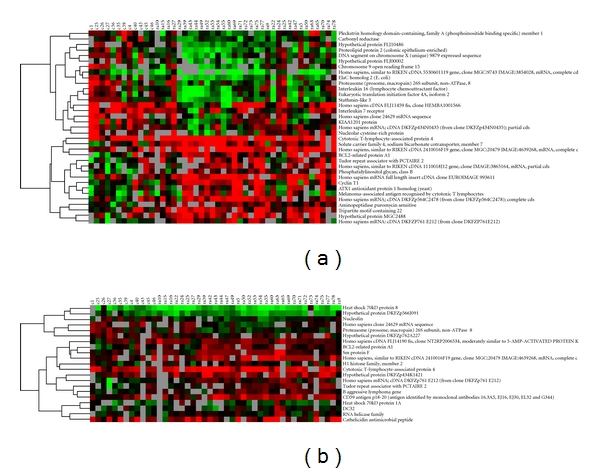
Cluster diagram of the expression of significantly expressed genes in 21 RA patients with the cold pattern, 12 RA patients with the heat pattern, and 12 healthy volunteers. Patients are indicated as vertical column headings, and gene symbols of transcripts are given in horizontal rows. Red represents a relative expression greater than the median expression level across all samples, and green represents an expression level lower than the median. (a) Significantly expressed genes between TCM cold-pattern RA patients and healthy control. (b) Significantly expressed genes between TCM heat-pattern RA patients and healthy control.

A comparison between RA patients with the cold pattern and the healthy control revealed 35 differentially regulated genes (Figure 1, Table 1). Among these differentially regulated genes, 16 were upregulated (fold ≥ 1.4) and 19 were downregulated (fold ≤ 1.4). 

An analysis of RA patients with the heat pattern versus the healthy control showed 21 differentially expressed genes (Figure 1, Table 2); 15 were upregulated (fold ≥ 1.4), and 6 were downregulated (fold ≤ 1.4) (Figure 1, Table 2).

Six genes showed the same pattern of differential expression between the cold and control groups and between the heat and control groups (Table 3): *MMGT1, TDRD7, GTF3C6, BCL2A1, CTLA4*, and *PSMD8*. Except for *PSMD8*, which was downregulated, these genes were upregulated in both the cold pattern and the heat pattern comparisons. 

### 3.2. Pathway Analysis Using DAVID

The KEGG pathway analyses of the significantly expressed genes using DAVID are shown in Tables 4, 5, and 6. The six genes shared between the cold pattern and the heat pattern comparisons were related to the following pathways (Table 4): *CAMs*, T-cell receptor signaling pathway, and the proteasome. *CTLA4* participates in the pathways of autoimmune thyroid disease, *CAMs*, and the T-cell receptor signaling and rheumatoid arthritis pathways. *PSMD8* is one subunit of the proteasome. These results showed that the RA patients with either the cold or heat pattern had the same five pathways, which were disordered compared to healthy persons. These were the common points in the pathways of the cold and heat pattern comparisons.

Compared to the heat pattern versus control analysis, the cold pattern versus control analysis revealed that different pathways were related to the following differentially expressed genes (Table 5): glycosylphosphatidylinositol (GPI) anchor biosynthesis, arachidonic acid metabolism, Jak-STAT signaling, hematopoietic cell lineage, primary immunodeficiency, cytokine-cytokine receptor interactions, ABC transporters, pentose and glucuronate interconversions, and axon guidance. These pathways, which were related to the differentially expressed genes, may be characteristic of the cold pattern.

The differentially expressed genes identified in the heat pattern versus control group analysis were related to the following pathways (Table 6): antigen processing and presentation, endocytosis, MAPK signaling, RNA degradation, hematopoietic cell lineage, complement and coagulation cascades, mTOR signaling, adipocytokine signaling, regulation of autophagy, and insulin signaling. These results may reveal characteristics of the heat pattern.

### 3.3. Results of Gene Ontology Analysis Using DAVID

The GO-discovered categories using DAVID analysis for similar and different significantly expressed genes between the cold pattern and the heat pattern comparisons are shown in Tables 7, 8, and 9. As shown in Table 7, the six shared differentially expressed genes of the cold and heat pattern comparisons were predominantly grouped into functional classes of protein binding and binding. *BCL2A1, CTLA4*, and *PSMD8* belong to biological processes of negative cellular regulation, and *TDRD7, GTF3C6*, and *PSMD8* are cellular constituents of intracellular organelle lumens. The results of these gene ontology analyses were common to the cold and heat patterns. 

Genes that were uniquely differentially expressed between the cold pattern and the control were predominantly grouped into functional classes of protein binding (18 genes) and binding (22 genes) (Table 8). A few genes belonged to interspecies interactions between organisms (*IL16, EIF4A2,* and *CCNT1*), multiorganism processes (*IL16, EIF4A2, CCNT1, TRIM22*), and the immune response (*IL16, IL7R, TRIM22, GBP3*). *SLC4A7* and *DCXR* are cellular constituents of the ribonucleoprotein complex. 

As shown in Table 9, the genes that were uniquely differentially expressed between the heat pattern and the control were predominantly grouped into functional binding classes, including nucleotide binding, ATP binding, adenyl ribonucleotide binding, adenyl nucleotide binding, purine nucleoside binding, nucleoside binding, unfolded protein binding, ribonucleotide binding, purine ribonucleotide binding, and purine nucleotide binding. A few genes belonged to the biological stress response process, the biotic stimuli response, the unfolded protein response, and the protein stimulus and cellular constituent response of the ribonucleoprotein complex.

The common point of GO-discovered categories in the cold and heat pattern comparisons was the cellular constituent of the ribonucleoprotein complex. The characteristic molecular function is protein binding in the cold pattern, whereas it is nucleoside and ribonucleotide binding in the heat pattern.

### 3.4. Gene Function Using GeneSpring Analysis

**Figure 2:**
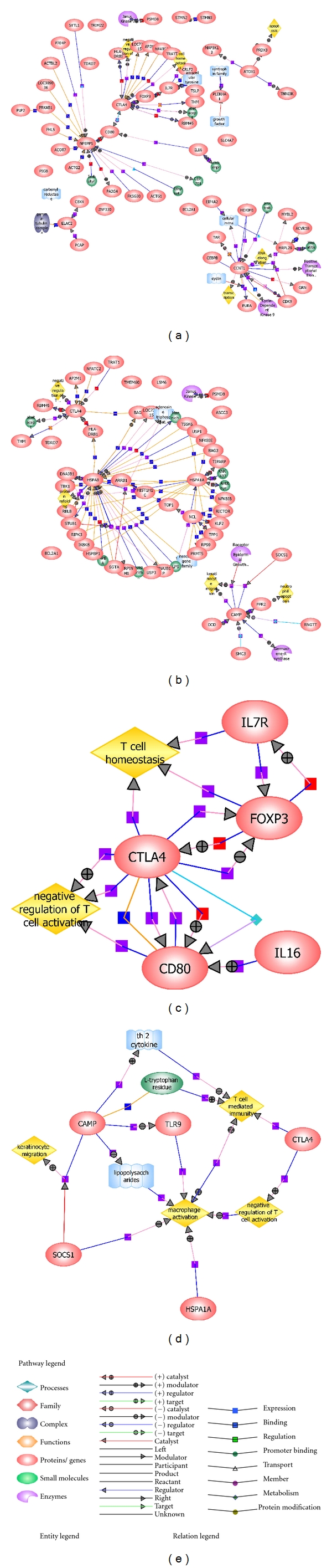
Networks of cold- and heat-pattern comparisons based on GeneSpring GX v 7.3.1 software. (a) Differently expressed genes between the cold pattern and the control. (b) Differently expressed genes between the heat pattern and the control. (c) Differently expressed genes between the cold pattern and the control in T-cell regulation. (d) Differently expressed genes between the heat pattern and the control in T-cell regulation.

The networks in Figure 2 were constructed using the GeneSpring GX v 7.3.1 software, as described in the Methods Section, starting with the lists of genes that were differently expressed between the cold pattern and the control (network A) and between the heat pattern and the control (network B). These networks revealed the functions and biological processes of the significantly expressed genes.

*CTLA4, PSMD8,* and *TDRD7* were in both the cold and heat pattern comparisons, similar to the *DAVID* analysis results. *CTLA4* and *CCNT1* are important centers of the cold pattern networks, and they cooperated with the *IL7R, IL16*, and *EIF4A2* proteins and participated in the negative regulation of T cells, T cell homeostasis, intracellular tyrosine, Janus Kinase 3, RNA elongation, and transcription. 

In the network of differently expressed genes between the heat pattern and the control, *CAMP, HSPA8,* and *HSPA1A* were highly connected with other differently expressed genes and were related to the negative regulation of T cells, protein refolding, keratinocyte migration, and neutrophil apoptosis.

Networks C and D revealed that the differently expressed genes in the cold and heat pattern comparisons were related to the regulation of T cells but in different ways. In the cold pattern, IL7R, CD80, and IL-6 processed the negative regulation of T-cell activity and T-cell homeostasis through *CTLA4*. However, in the heat pattern, except for *CTLA4,* which was related to the negative regulation of T cell activation, *CAMP, SOCS1, HSPA1A,* and *TLR7* participated in T-cell-mediated immunity, macrophage activation, and keratinocyte migration. 

### 3.5. Results of PPI Analysis

**Figure 3:**
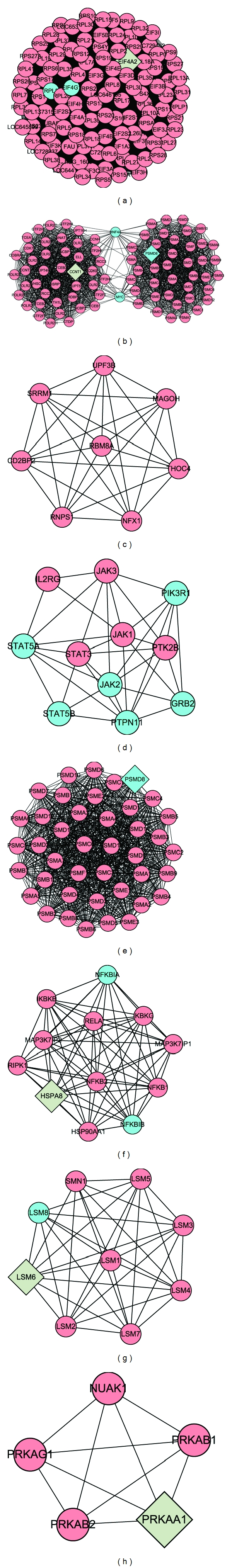
The PPI subnetworks based on the differently expressed genes made up of highly connected regions. (a–d): The subnetworks of the cold pattern versus control comparison. (e–h): The subnetworks of the heat pattern versus control comparison. Diamonds represent seed nodes. Cycles represent neighbor nodes. All edges represent interactions.

The PPI networks of the significantly expressed genes between the cold pattern and the control and between the heat pattern and the control are shown in Figure 3. There were four subnetworks for the cold-pattern comparison and four subnetworks for the heat patten comparison. The gene ontology analyses of each subnetwork are shown in Table 10. 

The PPI subnetworks of the cold-pattern versus control comparison (subnetworks A-D) revealed the seed nodes *EIFA2, CCNT1,* and *PSMD8* and their related proteins, which were similar to the differently expressed genes in the aformentioned analysis. The PPI subnetworks of the heat pattern versus control comparison (subnetworks E-H) revealed the seed nodes *PSMD8*, *HSPA8, LSM6,* and *PRKAB2*, which were also similar to the significantly differently expressed genes in the heat pattern and control comparison. The seed proteins and subnetworks related to the gene ontology analysis results were matched with the KEGG pathways, the gene ontology, and the functional analysis results using DAVID and GeneSpring based on the differently expressed genes in the cold- and heat-pattern comparisons (Table 10).

### 3.6. The Intersection Network between the Cold Pattern and the Heat Pattern

**Figure 4:**
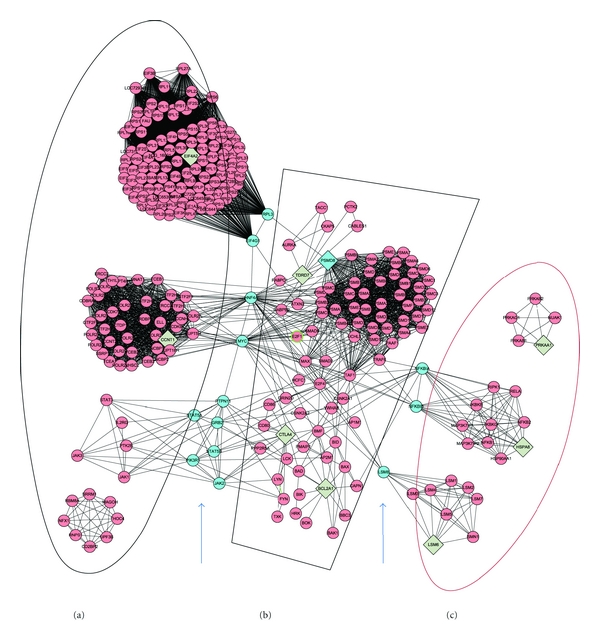
The PPI network of functional relationships between the cold and heat patterns. Diamonds represent seed nodes. Cycles represent neighbor nodes. All edges represent interactions. (a) Is the network specific to the cold pattern. (c) Is the network specific to the heat pattern. (b) Is the commonly shared network of both patterns, which is likely the common network of RA. The parts between (a) and (b) and between (b) and (c), as shown by arrows, are the connections between the highly condensed TCM pattern-related networks and the common shared network.

Figure 4 shows the intersection network between the cold pattern and the heat pattern, which includes the majority of differently expressed genes in the cold and heat pattern comparisons. All subnetworks are shown in Figures 3 and 4.

**Figure 5:**
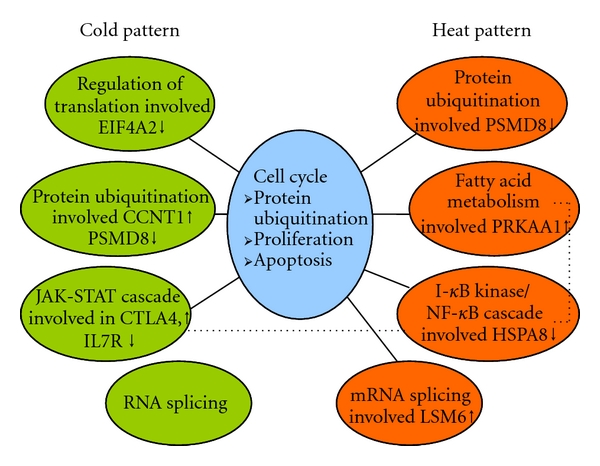
The gene ontology relationship between the TCM cold and heat patterns in RA. Cycles on the left represent cold-pattern-related biological processes. Cycles on the right represent heat-pattern-related biological processes. Cycles in the middle represent the biological processes common to the cold and heat patterns. Arrows represent the direction of the regulation of differently expressed genes: up signifies up-regulation, and down signifies down-regulation.

Based on the gene ontology analysis results (Table 10) and the other results, Figure 5 was drawn to outline the networks of the cold and heat patterns and to reveal the relationships between the two patterns in the functional and biological processes of the network.

In these networks, protein ubiquitination and RNA splicing were the common biological processes in the TCM cold and heat patterns of RA. The different biomarkers for the TCM cold and heat patterns of RA are obvious: the cold pattern was related to the regulation of translation and the *Jak-STAT* cascade, while the heat pattern was related to fatty acid metabolism and the I-*κ*B kinase/NF-*κ*B cascade. In addition, protein ubiquitination, proliferation, and apoptosis related to the cell cycle are the biological process connections between the cold pattern and the heat pattern. *CTLA4* and *PSMD8* were the significant biomarkers in both the cold and heat patterns. The significant biomarkers of the cold pattern were *EIF4A2, CCNT1,* and *IL7R*, while the significant biomarkers of the heat pattern were *PRKAA1, HSPA8,* and *LSM6*.

## 4. Discussion

In this study, we utilized software to identify network-based gene expression biomarkers and biomarkers that were organized by sets of differentially expressed genes that were members of established functional networks. Our major findings were the network-based gene expression biomarker pathways that were similar and different between the TCM cold-pattern RA patients and the TCM heat-pattern RA patients. 

The *CAMs*, T-cell receptor signaling pathway, and proteasome could be related to both the TCM cold and heat patterns in RA patients. Specifically, *CTLA4* (cytotoxic T lymphocyte antigen 4), which was a seed gene in these pathways and was up-regulated in both patterns, participates in the pathways of *CAMs* and T-cell receptor signaling. The *CTLA4* molecule is expressed on activated T lymphocytes and has recently been identified as an important negative regulator in autoimmune diseases. Quantitative alterations of *CTLA4 *contribute to autoimmune tissue destruction [43], and the expression of *CTLA4 *plays a downregulatory role in rheumatoid articular inflammation. *CTLA4* was also up-regulated on T cells in patients with RA, and the increase in *CTLA4* expression might exert a downregulation effect on tumor necrosis factor alpha and interleukin 1 beta production [44]. *CTLA4-Ig* in activated macrophages induces significant down-regulation in the cellular production of IL-6, TNF-alpha, IL1-beta, and TGF-beta for the treatment of RA [45]. In the GeneSpring analysis results, the functions of *CTLA4 *and its relationships with other immune molecules, such as *IL-16, IL-7R, FOXP3,* and *CD80*, which participates in T-cell homeostasis and the negative regulation of T-cell activation in cold-pattern RA patients, were identified. In the heat pattern RA patients, except for the negative regulation of T-cell activation, *CTLA4* participates in T-cell-mediated immunity, macrophage activation, and keratinocyte migration with the other seed genes, such as *CAMP, SOCS1, HSPA1A,* and *TLR9*. Our study showed that *CTLA4* is an important common negative regulator in both cold- and heat-pattern RA patients compared to healthy controls. 

*PSMD8* (Proteasome (prosome, macropain) 26S subunit, non-ATPase, 8), a significantly expressed gene in both cold- and heat-pattern RA patients, is one subunit of a protein-destroying apparatus that is involved in many essential cellular functions, such as the regulation of the cell cycle, cell differentiation, signal transduction pathways, antigen processing for appropriate immune responses, stress signaling, inflammatory responses, and apoptosis [46]. *PSMD8 *was down-regulated in both cold- and heat-pattern RA patients compared to healthy controls. The PPI analysis (Figure 3) showed that in the subnetwork B of cold-pattern RA patients and the subnetwork E of heat pattern RA patients, the *PSMD8*-related family showed similar biological functions; it was involved in the regulation of protein ubiquitination in the cell cycle. Therefore, in RA patients, the regulation of protein ubiquitination in the cell cycle is down-regulated in both cold- and heat-pattern patients. In PPI sub-networks C and G, a similar biological process, RNA splicing, was observed in both cold-pattern and heat-pattern RA patients. 

In TCM cold-pattern RA patients, pathways related to GPI anchor biosynthesis, arachidonic acid metabolism, *Jak-STAT* signaling, hematopoietic cell lineage, primary immunodeficiency, cytokine-cytokine receptor interaction, ABC transporters, pentose and glucuronate interconversions, and axon guidance were found. In these pathways, *CCNT1, IL7R, IL16,* and *EIF4A2* genes were included as the seeds. 

*CCNT1,* or *Cyclin T1*, is a protein in the highly conserved cyclin family, whose members are characterized by a dramatic periodicity in protein abundance throughout the cell cycle. *Cyclins* function as regulators of CDK kinases. Different cyclins exhibit distinct expression and degradation patterns, which contribute to the temporal coordination of each mitotic event. *Cyclin T1* is closely associated with *CDK9* kinase and is a major subunit of the transcription elongation factor p-TEFb. This cyclin and its kinase partner are involved in the phosphorylation and regulation of the carboxy-terminal domain (CTD) of the largest RNA polymerase II subunit [47]. *Cyclin T1* protein expression is highly regulated in CD4+ T cells and macrophages. *Cyclin T1* expression is low in resting CD4+ T cells that have been isolated from healthy donors, but upon T-cell activation, it is induced by a mechanism that involves posttranscriptional regulation [48, 49]. *Cyclin T1* expression is also low in freshly isolated monocytes, and it is up-regulated by a posttranscriptional mechanism within one to two days after the cells are cultured under conditions that allow for macrophage differentiation [50]. However, after one to two weeks in culture, *Cyclin T1* protein expression is shut off in macrophages by proteasome-mediated proteolysis. Treatment of macrophages with the immunosuppressive cytokine IL-10 accelerates this proteasome-mediated shut-off of *Cyclin T1* [51]. *Cyclin T1* can be reinduced by activation with agents, such as LPS, which indicates that the induction of *Cyclin T1* is a component of an innate immune response in mature macrophages [52, 53]. In our study, *CCNT1*, which was up-regulated, participated in RNA transcription and protein ubiquitination in cold-pattern RA patients, but no change was observed in heat-pattern RA patients. 

*IL7R* (Interleukin 7 receptor) participated in the *Jak-STAT* signaling pathway, hematopoietic cell lineage, primary immunodeficiency, and cytokine-cytokine receptor interactions in the KEGG pathway database for cold-pattern RA patients. The protein encoded by this gene is a receptor for interleukin 7 (IL7). The function of this receptor requires the interleukin 2 receptor gamma chain (*IL2RG*), a common gamma chain shared by the receptors of various cytokines, including interleukins 2, 4, 7, 9, and 15. This protein plays a critical role in the V (D) J recombination during lymphocyte development. This protein also controls the accessibility of the TCR gamma locus by *STAT5* and histone acetylation. Knockout studies in mice suggest that apoptosis blockade is an essential function of this protein during the differentiation and activation of T lymphocytes. The functional defects in this protein may be associated with the pathogenesis of severe combined immunodeficiency (*SCID*) [54]. NF-*κ*B-dependent gene expression in peripheral leukocytes is highly correlated with RA activity, as measured by the Disease Activity Score and C-reactive protein (*DAS28-CRP*). IL7R was one of the notably expressed associated with *DAS28-CRP* in the evaluation of the peripheral blood expression of genes regulated by *NF-*κ*B*, a key mediator of tumor necrosis factor-alpha (*TNF-alpha*) signaling, in patients with RA before and during treatment with anti-TNF-alpha or methotrexate (MTX). These results identify candidate markers, such as *IL7R*, that could lead to the development of a simple, minimally invasive pharmacodynamic assay for RA treatments directed at the *NF-*κ*B* pathway [55]. In our study, according to the GeneSpring analysis, *IL7R* was down-regulated and participated in T-cell regulation in cold-pattern RA patients but not in heat-pattern RA patients. In this case, *IL7R* can block apoptosis and promote the proliferation of CD4+ T cells in cold-pattern RA patients.

*IL16* (Interleukin 16, a lymphocyte chemoattractant factor) is a pleiotropic cytokine that functions as a chemoattractant, a modulator of T-cell activation, and an inhibitor of HIV replication. The signaling process of this cytokine is mediated by CD4. The product of this gene undergoes proteolytic processing, which yields two functional proteins. The cytokine function is exclusively attributed to the secreted C-terminal peptide, and the N-terminal product may play a role in cell cycle control. Caspase 3 is involved in the proteolytic processing of this protein. Alternate splicing results in multiple transcript variants [56]. Many studies have shown that *IL16* plays a role in the disease process underlying RA and joint destruction [57–59]. In our study, *IL16* was down-regulated and participated in T-cell regulation with *IL7R* in cold-pattern RA patients but not in heat pattern RA patients.

*EIF4A2* (eukaryotic translation initiation factor 4A2) is a gene for one of the protein-synthesis initiation factors involved in the binding of mRNA to the ribosome [60]. One study indicated that *EIF4A2* controls mRNA-specific translation and the protein synthesis rate in pancreatic beta-cells and that *EIF4A2* is down-regulated by high glucose levels in rat beta-INS832/13 cells [61]. In our study, according to the PPI analysis, *EIF4A2* as a seed gene was down-regulated in cold-pattern RA patients and was related to the regulation of translation and cell biosynthetic processes, but it was not altered in heat-pattern RA patients.

For TCM heat-pattern RA patients, closely related pathways, including antigen processing and presentation, endocytosis, *MAPK* signaling, RNA degradation, hematopoietic cell lineage, complement and coagulation cascades, mTOR signaling, adipocytokine signaling, regulation of autophagy, hypertrophic cardiomyopathy (*HCM*), and insulin signaling, were found. The differentially expressed major genes were *CAMP, PRKAA1, HSPA1A, HSPA8,* and *LSM6* in heat-pattern RA patients. 

*CAMP* (cathelicidin antimicrobial peptide) is an antimicrobial protein found in specific granules of polymorphonuclear leukocytes (PMNs) [62]. The cathelicidin family is characterized by a conserved N-terminal cathelin domain and a variable C-terminal antimicrobial domain that can be released from the precursor protein after cleavage by proteinases [63]. In our study, *CAMP* was up-regulated 2.33-fold and participated in T-cell regulation and cell proliferation in TCM heat-pattern RA patients but not in cold-pattern RA patients. 

*PRKAA1* (protein kinase, AMP-activated, alpha 1 catalytic subunit) belongs to the ser/thr protein kinase family. It is the catalytic subunit of the 5'-prime-AMP-activated protein kinase (*AMPK*). *AMPK* regulates the activities of a number of key metabolic enzymes through phosphorylation and protects cells from stresses that cause ATP depletion by switching off ATP-consuming biosynthetic pathways [64]. It participated in the mTOR signaling pathway, the adipocytokine signaling pathway, the regulation of autophagy, hypertrophic cardiomyopathy (*HCM*), and the insulin signaling pathway of the KEGG pathway in heat-pattern, RA patients but not in cold-pattern RA patients. According to the PPI analysis, *PRKAA1*, as a seed gene, was up-regulated and participated in fatty acid metabolism in TCM heat-pattern RA patients.

*HSPA1A* (heat shock 70 kDa protein 1A) is a 70 kDa heat shock protein and a member of the heat shock protein 70 family. In conjunction with other heat shock proteins, this protein stabilizes existing proteins against aggregation and mediates the folding of newly translated proteins in the cytosol and in organelles. It is also involved in the ubiquitin-proteasome pathway through interaction with the AU-rich element RNA-binding protein 1. It is located in the major histocompatibility complex class III region in a cluster with two closely related genes that encode similar proteins [65]. According to the GeneSpring analysis results, *HSPA1A* was up-regulated and participated in spliceosome, antigen processing and presentation, endocytosis, *MAPK* signaling pathway, and prion diseases using the KEGG pathway and in T-cell regulation in heat pattern RA patients. Based on the PPI analysis results, it also participated in the *I-*κ*B kinase/NF-*κ*B* cascade in heat pattern RA patients. *HSPA8* (heat shock 70 kDa protein 8) belongs to the heat shock protein 70 family, which contains both heat-inducible and constitutively expressed members. The latter members are called heat-shock cognate proteins. This gene encodes a heat-shock cognate protein, which binds to nascent polypeptides to facilitate correct folding. It also functions as an ATPase in the disassembly of clathrin-coated vesicles during the transport of membrane components through the cell [66]. Similar to *HSPA1A, HSPA8* also participated in the spliceosome, antigen processing and presentation, endocytosis, and *MAPK* signaling pathway in heat-pattern RA patients. According to the PPI analysis results, *HSPA8*, as a seed gene, was down-regulated and also participated in the *I-*κ*B kinase/NF-*κ*B* cascade in TCM heat-pattern RA patients. *LSM6* is related to Sm-like proteins, which form a stable heteromer that is present in tri-snRNP particles, and is important for pre-mRNA splicing [67]. According to the KEGG pathway, mRNA splicing, and PPI analysis results, *LSM6*, as a seed gene, was up-regulated and participated in the spliceosome and RNA degradation in TCM heat-pattern RA patients.

We outlined the gene ontology relationship between the TCM cold and heat patterns in RA patients (Figure 5) by first combining the PPI results of interactions between TCM cold and heat patterns (Figure 4) and then combining them with all other data. In this network, common genes and biological processes and significantly different gene-based pathways and biological processes between the TCM cold- and heat-pattern patients were identified, and these can be used as biomarkers for the classification of the two TCM RA patterns. In the network, protein ubiquitination and RNA splicing were the common biological processes in the TCM cold and heat patterns. The different biomarkers for the TCM cold and heat patterns were obvious: the TCM cold pattern was related to the regulation of translation and the *Jak-STAT* cascade, and the TCM heat pattern was related to fatty acid metabolism and the *I-*κ*B kinase/NF-*κ*B* cascade. In addition, protein ubiquitination, proliferation and apoptosis related to the cell cycle may be the connecting biological processes between the TCM cold and heat patterns. *CTLA4* and *PSMD8* were the same significant biomarkers in both the TCM cold and heat patterns. The significant biomarkers for the TCM cold pattern could be *EIF4A2, CCNT1,* and *IL7R*, and the significant biomarkers for the TCM heat pattern could be *PRKAA1, HSPA8,* and *LSM6*.

This study has some limitations, including the lack of validation by PCR or Western blotting or other clinical analysis approaches for the microarray data. Because TCM pattern classification is based on an assemblage of multiple clinical manifestations, including clinical signs and symptoms and tongue and pulse appearances, the relevant biomedical networks are likely equally complex. Thus, various genes and complicated interactions were included in the results, which makes short-term validation of the results difficult. However, in this study, we use gene interaction analysis software (IPA) to represent functional clusters of different genes. Based on these findings, additional Omic (metabolics and proteomics) studies have been designed to validate the functional clusters. In addition, a more optimal study design, which includes detailed aspects such as female menstruation, diet, and activity controls, is warranted. 

## 5. Conclusions

EIF4A2, CCNT1, and IL7R, which were related to the up-regulation of cell proliferation and the Jak-STAT cascade, were significant gene biomarkers of the TCM cold pattern of RA. PRKAA1, HSPA8, and LSM6, which were related to fatty acid metabolism and the I-*κ*B kinase/NF-*κ*B cascade, were significant biomarkers of the TCM heat pattern of RA. The network-based gene expression biomarkers for the TCM cold and heat patterns may be helpful for the further stratification of RA patients when deciding on interventions or clinical trials. 

## Figures and Tables

**Table 1:** Differentially expressed genes in cold-pattern RA patient versus healthy control.

Gene name	Definition	Accession no.	Cold/control ratio
*Upregulated gene (>1.4)*			
* MMGT1*	Membrane magnesium transporter 1	AL157477	1.70
* TDRD7*	Tudor domain containing 7	AB025254	1.56
* SLC4A7*	Solute carrier family 4, sodium bicarbonate Cotransporter, member 7	AF047033	1.54
* MRPL28*	Mitochondrial ribosomal protein L28	NM_006428	1.54
* CCNT1*	Cyclin T1	NM_001240	1.52
* GTF3C6*	General transcription factor IIIC, polypeptide 6, alpha 35 kDa	BC011593	1.49
* BCL2A1*	BCL2-related protein A1	NM_004049	1.45
* TRIM22*	Tripartite motif-containing 22	NM_006074	1.45
* CTLA4*	Cytotoxic T-lymphocyte-associated protein 4	AF414120	1.45
* KLRAQ1*	KLRAQ motif containing 1	BC011978	1.45
* GBP3*	Guanylate binding protein 3	AL136680	1.43
* MIS12*	MIS12, MIND kinetochore complex component, homolog (*S. pombe*)	NM_024039	1.43
* PDCD2*	Programmed cell death 2	AJ420535	1.43
* ATOX1*	ATX1 antioxidant protein 1 homolog (yeast)	NM_004045	1.43
* PIGB*	Phosphatidylinositol glycan, class B	NM_004855	1.41
* NPEPPS*	Aminopeptidase puromycin sensitive	NM_006310	1.41
*Downregulated gene (<1 : 1.4)*			
* Unknown*	Homo sapiens cDNA FLJ11459 fis, clone HEMBA1001566	AK021521	0.59
* Unknown*	Homo sapiens mRNA; cDNA DKFZp434I138 (from clone DKFZp434I138)	AL122121	0.66
* Unknown*	Homo sapiens clone 24629 mRNA sequence	AF052160	0.66
* ZNF330*	Zinc finger protein 330	NM_014487	0.67
* PLP2*	Proteolipid protein 2 (colonic epithelium-enriched)	NM_002668	0.68
* PTGES2*	Prostaglandin E synthase 2	NM_025072	0.68
* PSMD8*	Proteasome (prosome, macropain) 26S subunit, non-ATPase, 8	NM_002812	0.68
* IL7R*	Interleukin 7 receptor	NM_002185	0.68
* STMN3*	Stathmin-like 3	NM_015894	0.68
* ABCC10*	ATP-binding cassette, sub-family C (CFTR/MRP), member 10	AK000002	0.69
* ELAC2*	ElaC homolog 2 (*E. coli*)	NM_018127	0.69
* PLEKHA1*	Pleckstrin homology domain-containing, family A (phosphoinositide binding specific) member 1	NM_021622	0.69
* DCXR*	Dicarbonyl/L-xylulose reductase	NM_016286	0.70
* EIF4A2*	Eukaryotic translation initiation factor 4A2	NM_001967	0.70
* IL16*	Interleukin 16 (lymphocyte chemoattractant factor)	M90391	0.70
* GRAMD1B*	GRAM domain containing 1B	AB033027	0.71
* LAGE3*	L antigen family, member 3	NM_006014	0.71
* TMEM80*	Transmembrane protein 80	BC008671	0.71
* SLIT3*	Slit homolog 3 (Drosophila)	AL122074	0.71

**Table 2:** Differentially expressed genes in heat-pattern RA patient versus healthy control.

Gene name	Definition	Accession no.	Heat/control ratio
*Up-regulated gene (>1.4)*			
* CAMP*	Cathelicidin antimicrobial peptide	NM_004345	2.33
* MMGT1*	Membrane magnesium transporter 1	AL157477	1.69
* TDRD7*	Tudor domain containing 7	AB025254	1.56
* LSM6*	LSM6 homolog, U6 small nuclear RNA associated (*S. cerevisiae*)	NM_007080	1.54
* CCDC55*	Coiled-coil domain containing 55	NM_032141	1.54
* GTF3C6*	General transcription factor IIIC, polypeptide 6, alpha 35 kDa	BC011593	1.54
* CD59*	CD59 molecule, complement regulatory protein	NM_000611	1.52
* CTLA4*	Cytotoxic T-lymphocyte-associated protein 4	AF414120	1.52
* ASCC3*	Activating signal cointegrator 1 complex subunit 3	AY013288	1.47
* PRKAA1*	Protein kinase, AMP-activated, alpha 1 catalytic subunit	AK024252	1.45
* BCL2A1*	BCL2-related protein A1	NM_004049	1.45
* TMEM60*	Transmembrane protein 60	NM_032936	1.43
* HSPA1A*	Heat shock 70 kD protein 1A	NM_005345	1.43
* PARP9*	Poly (ADP-ribose) polymerase family, member 9	NM_031458	1.43
* HIST1H1C*	histone cluster 1, H1c	NM_005319	1.43
*Down-regulated gene (<1 : 1.4)*			
* HSPA8*	Heat shock 70 kD protein 8	NM_006597	0.63
* SLC43A3*	Solute carrier family 43, member 3	NM_014096	0.67
* Unknown*	Homo sapiens clone 24629 mRNA sequence	AF052160	0.67
* PSMD8*	Proteasome (prosome, macropain) 26S subunit, non-ATPase, 8	NM_002812	0.69
* NCL*	Nucleolin	NM_005381	0.70
* LBH*	Limb bud and heart development homolog (mouse)	NM_030915	0.71

**Table 3:** The same significantly expressed genes found in the differences between the cold and control groups and between the heat and control groups.

Gene name	Definition	Accession no.	Ratio in cold versus control	Ratio in heat versus control
*MMGT1*	Membrane magnesium transporter 1	AL157477	1.70	1.69
*TDRD7*	Tudor domain containing 7	AB025254	1.56	1.56
*GTF3C6*	General transcription factor IIIC, polypeptide 6, alpha 35 kDa	BC011593	1.49	1.54
*BCL2A1*	BCL2-related protein A1	NM_004049	1.45	1.45
*CTLA4*	Cytotoxic T-lymphocyte-associated protein 4	AF414120	1.45	1.52
*PSMD8*	Proteasome (prosome, macropain) 26S subunit, non-ATPase, 8	NM_002812	0.68	0.69

**Table 4:** The pathways shared by the differently expressed genes of the cold pattern versus control and heat pattern versus control comparisons.

KEGG pathway	Related genes	Ratio in cold versus control	Ratio in heat versus control
hsa05320: Autoimmune thyroid disease	*CTLA4*	1.45	1.52
hsa04514: Cell adhesion molecules (CAMs)	*CTLA4*	1.45	1.52
hsa04660: T cell receptor signaling pathway	*CTLA4*	1.45	1.52
hsa05323: Rheumatoid arthritis	*CTLA4*	1.45	1.52
hsa03050: Proteasome	*PSMD8*	0.68	0.69

**Table 5:** The pathways related to the differently expressed genes between the cold pattern and control comparison.

KEGG pathway	Related genes	Ratio
hsa00563: Glycosylphosphatidylinositol (GPI) anchor biosynthesis	*PIGB*	1.41
hsa00590: Arachidonic acid metabolism	*PTGES2*	0.68
hsa04630: Jak-STAT signaling pathway	*IL7R*	0.68
hsa04640: Hematopoietic cell lineage	*IL7R*	0.68
hsa05340: Primary immunodeficiency	*IL7R*	0.68
hsa04060: Cytokine-cytokine receptor interaction	*IL7R*	0.68
hsa02010: ABC transporters	*ABCC10*	0.69
hsa00040: Pentose and glucuronate interconversions	*DCXR*	0.70
hsa04360: Axon guidance	*SLIT3*	0.71

**Table 6:** The pathways related to the differently expressed genes between the heat pattern and control comparison.

KEGG pathway	Related genes	Ratio
hsa03040: Spliceosome	*LSM6, HSPA1A, HSPA8*	1.54, 1.43, 0.63
hsa04612: Antigen processing and presentation	*HSPA1A, HSPA8*	1.43, 0.63
hsa04144: Endocytosis	*HSPA1A, HSPA8*	1.43, 0.63
hsa04010: MAPK signaling pathway	*HSPA1A, HSPA8*	1.43, 0.63
hsa03018: RNA degradation	*LSM6*	1.54
hsa04640: Hematopoietic cell lineage	*CD59*	1.52
hsa04610: Complement and coagulation cascades	*CD59*	1.52
hsa04150: mTOR signaling pathway	*PRKAA1*	1.45
hsa04920: Adipocytokine signaling pathway	*PRKAA1*	1.45
hsa04140: Regulation of autophagy	*PRKAA1*	1.45
hsa05410: Hypertrophic cardiomyopathy (HCM)	*PRKAA1*	1.45
hsa04910: Insulin signaling pathway	*PRKAA1*	1.45
hsa05020: Prion diseases	*HSPA1A*	1.43
hsa05130: Pathogenic Escherichia coli infection	*NCL*	0.70

**Table 7:** GO-discovered categories for the same expressed genes in the cold versus control and heat versus control groups.

GO ID	GO name	Number of genes	*P* value	Gene name
*Biological process*				
GO:0048523	Negative regulation of cellular process	3	0.1085	*BCL2A1, CTLA4, PSMD8*
GO:0048519	Negative regulation of biological process	3	0.1264	*BCL2A1, CTLA4, PSMD8*
*Cellular constituent*				
GO:0070013	Intracellular organelle lumen	3	0.0993	*TDRD7, GTF3C6, PSMD8*
*Molecular function*				
GO:0005515	Protein binding	5	0.2392	*TDRD7, BCL2A1, GTF3C6, CTLA4, PSMD8*
GO:0005488	Binding	6	0.3880	*MMGT1, TDRD7, BCL2A1, GTF3C6, CTLA4, PSMD8*

**Table 8:** GO-discovered categories for the genes that differed between the cold pattern and the control.

GO ID	GO name	Number of genes	*P* value	Gene name
*Biological process*				
GO:0044419	Interspecies interaction between organisms	3	0.0600	*IL16, EIF4A2, CCNT1*
GO:0051704	Multiorganism process	4	0.0692	*IL16, EIF4A2, CCNT1, TRIM22*
GO:0006955	Immune response	4	0.0714	*IL16, IL7R, TRIM22, GBP3*
*Cellular constituent*				
GO:0005902	Ribonucleoprotein complex	2	0.0508	*SLC4A7, DCXR*
*Molecular function*				
GO:0005515	Protein binding	18	0.0210	*PLP2, ELAC2, PTGES2, IL16, STMN3, ATOX1, CCNT1, NPEPPS, IL7R, TRIM22, MIS12, ZNF330, PDCD2, SLIT3, LAGE3, EIF4A2, SLC4A7, DCXR*
GO:0005488	Binding	22	0.0866	*PLP2, ELAC2, PTGES2, IL16, STMN3, ATOX1, CCNT1, ABCC10, NPEPPS, IL7R, TRIM22, MIS12, ZNF330, PDCD2, SLIT3, MRPL28, LAGE3, EIF4A2, SLC4A7, GBP3, DCXR, PLEKHA1*

**Table 9:** GO-discovered categories for the genes that differed between the heat pattern and the control.

GO ID	GO name	Number of genes	*P* value	Gene name
*Biological process*				
GO:0006950	Response to stress	5	0.0235	*CAMP, CD59, PRKAA1, HSPA1A, HSPA8*
GO:0009607	Response to biotic stimulus	3	0.0287	*CAMP, HSPA1A, HSPA8*
GO:0006986	Response to unfolded protein	2	0.0492	*HSPA1A, HSPA8*
GO:0051789	Response to protein stimulus	2	0.0733	*HSPA1A, HSPA8*
*Cellular constituent*				
GO:0030529	Ribonucleoprotein complex	4	0.0076	*LSM6, HSPA1A, NCL, HSPA8*
*Molecular function*				
GO:0000166	Nucleotide binding	5	0.0205	*ASCC3, PRKAA1, HSPA1A, NCL, HSPA8*
GO:0005524	ATP binding	4	0.0357	*ASCC3, PRKAA1, HSPA1A, HSPA8*
GO:0032559	Adenyl ribonucleotide binding	4	0.0369	*ASCC3, PRKAA1, HSPA1A, HSPA8*
GO:0030554	Adenyl nucleotide binding	4	0.0423	*ASCC3, PRKAA1, HSPA1A, HSPA8*
GO:0001883	Purine nucleoside binding	4	0.0440	*ASCC3, PRKAA1, HSPA1A, HSPA8*
GO:0001882	Nucleoside binding	4	0.0447	*ASCC3, PRKAA1, HSPA1A, HSPA8*
GO:0051082	Unfolded protein binding	2	0.0592	*HSPA1A, HSPA8*
GO:0032553	Ribonucleotide binding	4	0.0623	*ASCC3, PRKAA1, HSPA1A, HSPA8*
GO:0032555	Purine ribonucleotide binding	4	0.0623	*ASCC3, PRKAA1, HSPA1A, HSPA8*
GO:0017076	Purine nucleotide binding	4	0.0695	*ASCC3, PRKAA1, HSPA1A, HSPA8*

**Table 10:** The gene ontology analysis of the cold pattern versus control and the heat pattern versus control comparisons.

GO ID	Description	*P *value
	Cold pattern versus control	

*Subnetworks A*		
6455	Translational elongation	4.26E-168
6416	Translation	6.96E-146
44249	Cell biosynthetic process	1.87E-104
9059	Macromolecule biosynthetic	8.699E-70
10467	Gene expression	1.156E-68
*Subnetworks B*		
31145	Anaphase-promoting complex-dependent proteasomal ubiquitin-depended protein catabolic process	4.326E-81
51436	Negative regulation of ubiquitin protein ligase activity during mitotic cell cycle	4.326E-81
51352	Negative regulation of ligase activity	1.235E-80
51444	Positive regulation of ligase activity during mitotic cell cycle	1.235E-80
51437	Positive regulation of ligase activity	9.253E-80
*Subnetworks C*		
6374	Nuclear mRNA splicing, via spliceosome	9.582E-17
375	RNA splicing, via transesterification reactions	9.582E-17
377	RNA splicing, via transesterification reactions with bulged adenosine as nucleophile	9.582E-17
6395	RNA splicing	1.284E-14
6397	mRNA process	2.703E-14
*Subnetworks D*		
7259	JAK-STAT cascade	2.231E-09
19221	Cytokine and chemokine mediated signaling pathway	1.603E-08
1553	Luteinization	5.211E-07
1779	Natural killer cell differentiation	5.211E-07
46544	Development of secondary male sexual characteristics	5.211E-07

	Heat pattern versus control	

*Subnetworks E*		
31145	Anaphase-promoting complex-dependent proteasomal ubiquitin-depended protein catabolic process	1.45E-105
51436	Negative regulation of ubiquitin protein ligase activity during mitotic cell cycle	1.45E-105
51352	Negative regulation of ligase activity	4.16E-105
51444	Positive regulation of ligase activity during mitotic cell cycle	4.16E-105
51437	Positive regulation of ligase activity	3.14E-104
*Subnetworks F*		
51101	Regulation of DNA binding	1.508E-05
51098	Regulation of binding	3.286E-05
43123	Positive regulation of I-*κ*B kinase/NF-*κ*B cascade	5.187E-05
9607	Response to biotic stimulus	5.593E-05
43122	Regulation of I-*κ*B kinase/NF-*κ*B cascade	6.457E-05
*Subnetworks G*		
6395	RNA splicing	1.284E-14
6397	mRNA splicing	2.703E-14
16071	mRNA metabolic process	7.908E-14
6394	RNA processing	1.458E-12
16070	RNA metabolic process	1.175E-10
*Subnetworks H*		
6633	Fatty acid biosynthetic process	1.498E-09
46394	Carboxylic acid biosynthetic process	2.761E-09
16053	Organic acid biosynthetic process	2.761E-09
6631	Fatty acid metabolic process	7.384E-08
32787	Monocarboxylic acid metabolic process	2.767E-07
